# “*I had a feeling the period did not have an opening to come out:”* A qualitative assessment of Type III Female Genital Mutilation/Cutting and menstrual health among Somali communities in Kenya

**DOI:** 10.3389/fgwh.2026.1753459

**Published:** 2026-03-25

**Authors:** Anne Achieng, Sarah C. Blake, Ramatou Ouedraogo, Caroline Kabiru, Marni Sommer

**Affiliations:** 1 African Population and Health Research Center, Nairobi, Kenya; 2 Department of Sociomedical Sciences, Mailman School of Public Health, Columbia University, New York, NY, United States

**Keywords:** East Africa, female genital mutilation/cutting, infibulation, Kenya, menstrual health, menstruation, qualitative research methods

## Abstract

**Introduction:**

Type III female genital mutilation/cutting (FGM/C), or infibulation, negatively impacts girls' and women's health and well-being. Some evidence suggests women who have experienced infibulation may have distinct menstrual health challenges. However, the intersection of infibulation and menstrual health is under-explored. The present study documented women's and providers' perspectives on menstrual health and infibulation among Somali communities in Wajir County, Kenya.

**Methods:**

We conducted a qualitative study including in-depth interviews (*n* = 23) with Somali women ages 18–45, and key informant interviews (*n* = 10) with healthcare providers. Interviews with women explored their reflections on and experiences of FGM/C, including infibulation, menstrual health, and perceived connections between the two. Interviews with healthcare providers explored their perspectives on women's healthcare needs in relation to menstrual health and infibulation.

**Results:**

Thematic analysis identified three themes: (1) informal discussion among women shapes their menstruation and infibulation knowledge; (2) multiple factors that shape women's care-seeking for menstrual health concerns; and (3) healthcare providers lack the necessary preparation to address menstrual health concerns among women who have undergone infibulation.

**Discussion:**

This study's findings provide insights into the intersections of menstrual health and infibulation within the broader educational, social and healthcare context of Somali communities in rural Kenya. While women learn, interpret, and describe infibulation as a source of acute menstrual health problems, including menstrual pain and irregular bleeding, clinical evidence on this relationship is needed. Findings illuminated multiple factors inhibiting Somali women's receipt of adequate care for menstrual health needs. Additional challenges included women's perceptions of healthcare quality, distrust of formal healthcare providers, and gendered family decision-making processes around healthcare. Healthcare providers' inadequate training and support hindered diagnosis and care for menstrual disorders for women who have and have not experienced infibulation, while confusion about Kenya's FGM/C ban further complicates provider engagement.

## 1 Introduction

Globally, more than 200 million girls and women are estimated to have undergone female genital mutilation/cutting (FGM/C) (1). The World Health Organization (WHO) delineates four primary types of FGM/C, all of which involve removing or closing off portions of the external female reproductive organs (World Health Organization, 2025). FGM/C has been associated with immediate and long-term health problems, such as excessive bleeding, scarring, urinary tract infections, mental health disorders, and loss of sexual feeling (2–4). Infibulation (Type III) involves closing of the labia, leaving only a small hole for urine and menstrual fluid to pass (5). Among girls and women who have experienced FGM/C, an estimated 10 percent have undergone Type III, or infibulation (1). Evidence suggests that women who have experienced infibulation may face distinct menstrual health challenges (4, 6). However, women's and providers' perspectives on the relationship between Type III FGM/C and menstrual health have not been adequately explored.

FGM/C practices vary widely by community, and are rooted in collective beliefs about women's sexual purity in maintaining family honor, including their virginity as a requisite for marriageability (7–10). FGM/C is nearly ubiquitous among Somali communities in East Africa (2, 11, 12). While infibulation, known as “Pharaonic” or “firuani” is the most common type, another form, known as “sunna” or “sunni” (referred to as “sunni” here), accounts for a substantial and possibly increasing share of the practice (12–14). Although generally considered to be less invasive, the prevalence and health impacts of the sunni form are difficult to determine (2, 14–16). The term can also describe varied practices, which may not align with the WHO taxonomy, further complicating efforts to track changes in the prevalence of FGM/C by type (16, 17).

In general, Kenya's national policies frame FGM/C as a form of gender-based violence. Specific laws criminalize all forms of FGM/C as a “harmful traditional practice” and prohibit healthcare workers from participating in any procedures, including re-infibulation (18–21). At the same time, health policy seeks to ensure that women and girls who have undergone FGM/C can access appropriate healthcare to address the complications of FGM/C, primarily in relation to reducing risks related to childbirth (7, 22). Existing policy and provider guidance do not specifically address menstrual blood flow and/or pain in relation to FGM/C. In addition, as in other contexts, healthcare providers may lack expertise to identify distinct types of FGM/C, nor interpret how they impact women's health (12, 15, 16).

Menstrual health is defined as “a state of complete physical, mental, and social well-being and not merely the absence of disease or infirmity, in relation to the menstrual cycle” (23). It includes access to accurate and timely education; adequate menstrual management materials; appropriate medical diagnosis and care for menstrual disorders; and a supportive social and physical environment. International guidance highlights menstrual health problems as among the distinct issues that healthcare providers should be prepared to address in caring for women who have experienced FGM/C, including infibulation (5). Yet research on FGM/C's impact on menstrual health is inconsistent. Some studies suggest infibulation carries distinct negative menstrual health effects, while others find little or no such association (4, 24–27).

Due to a lack of research on menstrual health in general, there is only limited evidence on the prevalence of conditions such as severe menstrual pain (dysmenorrhea), and irregular bleeding among girls and women globally (28–30). Evidence is particularly weak in low- and middle-income country contexts, including Kenya (28, 29, 31). Improved evidence on women's experiences of challenging menstrual symptoms may contribute to improvements in care across diverse global contexts.

The present study aimed to contribute to building the evidence base on FGM/C, including infibulation, and its relationship to menstrual health. We explored both women's and healthcare providers' perspectives on the ways in which menstruation and infibulation may intersect; women's related healthcare seeking behaviors; and their recommendations for improving interactions with the formal health system.

## 2 Materials and methods

This qualitative research study utilized two methods: (1) Key Informant Interviews (KIIs) with healthcare providers who interact with Somali women around reproductive and menstrual health; and (2) In-Depth Interviews (IDIs) with Somali women aged 18–49 in a context where infibulation is common.

### 2.1 Ethical review

The study received ethical approval from Amref Health Africa Ethics and Scientific Review Committee in Kenya and Columbia University Irving Medical Center Institutional Review Board in the United States.

### 2.2 Research setting

This study was conducted in Wajir County in northeast Kenya, a rural region with a population of approximately 800,000 who are primarily ethnic Somali Muslim pastoralists (32). More than 95 percent of women ages 15–49 in Wajir County have experienced some form of FGM/C, and unlike other areas of Kenya, this rate has not declined over time (33, 34). Wajir has been the site of multiple programmatic efforts to reduce FGM/C. These have included alternative rites of passage for girls; engagement with religious leaders to address religious arguments for the practice; and campaigns involving health providers to build awareness of the practice's health effects (7, 34, 35).

### 2.3 Sampling and recruitment

For the KIIs (*n* = 10), we purposively sampled diverse healthcare providers in clinics and referral facilities who provide care to women who have experienced infibulation (see Table 1). Key informants ranged in age from mid-20s to late 50s. For the IDIs (*n* = 23), we used convenience and snowball sampling in healthcare facilities to identify women ages 18–49 who were from Somali communities where Type III FGM/C is common (see Table 2). Healthcare providers informed women about the project, and interested women were connected to the research team. The researchers also invited participants to refer other women in the community. Researchers then contacted the women by phone call or text to explore their interest. Due to the illegality of FGM/C in Kenya, the research team did not screen women for FGM/C status or type.

**Table 1:** Overview of Key informant sample.

	Gender		Total
	Female	Male	
Role			
Doctor or Medical Officer	2		
Nurse	5	2	
Traditional birth attendant	1	0	
Total	8	2	10

**Table 2:** Overview of in-depth interview sample.

Age	Marital status		Total
	*Ever married*	*Never married*	
Age 18–25	0	10	
Age 26–34	6	1	
Age 35–43	6	0	
Total	12	11	23

Sampling for the in-depth interviews followed a saturation approach (36). Although the initial target was 25 women, recruitment was concluded at 23 participants once data saturation was reached. Saturation was determined through regular debriefing sessions with research assistants, during which we evaluated emerging themes and identified where experiences and perceptions had begun to stagnate or lacked further variation.

### 2.4 Data collection

Two female research assistants, H.A. and A.A., conducted the key informant interviews and in-depth interviews between January and February 2023. Both research assistants were from Wajir, fluent in Somali, Swahili, and English. As part of the training and preparation for the study, the research team engaged in reflexivity exercises around their own experiences of menstruation and FGM/C prior to data collection and reflected on the positionality of all members of the team, internal and external to Kenya, in relation to study participants. The larger research team, including the co-Principal Investigators (co-PIs) and research staff, provided training and oversight.

Key informant interviews were conducted primarily in English, with two in Somali. Interviews were held in a confidential space within a clinic or hospital setting. In-depth interviews were held in a location chosen by the participant, either at home or an alternative confidential space. Based on women's preferences, interviews were conducted in Swahili or Somali, with the majority in Somali. Both types of interviews ranged from 30 to 60 min, were audio-recorded, and then translated and transcribed into English for analysis.

#### 2.4.1 Key informant interviews (KII)

We used a semi-structured interview guide to explore providers' experiences caring for women who had undergone infibulation, focusing on menstrual health concerns; their perspectives on the social and health dimensions of infibulation and menstrual health, and how this might affect women's healthcare seeking behavior and wellbeing. Interviews also addressed providers' training on clinical care for women who have undergone infibulation, and their recommendations for improving care. All participants provided informed written consent before beginning.

#### 2.4.2 In-depth interviews (IDI)

We used a semi-structured interview guide to explore women's views and experiences of FGM/C and menstruation. Topics included their knowledge and learning around menstrual health and infibulation; perceptions of menstrual health challenges that for women and girls who have undergone infibulation; related healthcare seeking behaviors; and their recommendations for addressing the menstrual needs of girls and women who have experienced infibulation. All participants provided verbal informed consent prior to interviews.

### 2.5 Data analysis

We used a three step practical thematic analysis approach (37) that began with a reflexive reading of transcripts and immersion in the data. The research team then followed a collaborative process of reflecting on and engaging with the data, observing information and experiences and narratives relevant to the research objectives. Two researchers drafted a codebook representing a combination of predefined codes reflecting topics of interest and codes emerging from the data (37, 38). The entire research team reviewed and provided inputs into the draft codebook. Using Dedoose software, two members of the research team then conducted initial coding, and a third member completed a second round of coding. After reviewing coded data, the research team collaboratively drafted emerging themes.

## 3 Results

We identified three themes related to the intersection of menstrual health and infibulation (see Table 3**)**: (1) informal discussion shapes menstruation and infibulation knowledge; (2) multiple factors shape women's care-seeking for menstrual concerns; (3) healthcare providers lack the necessary preparation to address menstrual health concerns among women who have undergone infibulation. No meaningful differences in responses emerged in relation to women's age or marital status, nor the professional position, gender, and age of the healthcare providers.

**Table 3:** Results and example quotes.

Theme	Dimensions	Example Quotes
Informal Discussion Shapes Menstruation and Infibulation Knowledge	Women experienced FGM/C as painful and surprising and received little guidance on menstruation prior to menarche.	*I thought I had been pricked by a thorn (on first seeing menstrual blood)*
	Women learned from family and community members that infibulation caused acute pain and irregular menstrual flow.	I remember a lady (female health provider) telling me that the wound had closed up further (after infibulation) and if she could cut it open…it would feel better. I had a feeling the period did not have an opening to come out from, and all the filth remained inside me.
	Some women invoked the idea that infibulation contributed to worse menstrual health symptoms as a reason to oppose the practice in favor of other forms of FGM/C.	People say the traditional way of cutting…the girls (were) disturbed during menstruation, some girls faint…but for sunni girls…menstruation is not disturbing.
Multiple factors shape care-seeking for menstrual concerns	Disagreements surrounded the question of whether adolescent girls had more severe menstrual problems than older women or whether other factors drove healthcare decision-making.	*There are those* (infibulated women) *who after being married they have menstruation every month. There are those* (who) *even after giving birth it is still painful.*…*their bodies are returning to the circumcised way; it stays with them.*…*If they go to the hospital*
	Intergenerational dynamics influenced women's sources of healthcare. Tensions surrounded decisions about whether younger women should use traditional or formal healthcare services.	*I wasn't getting better with those herbal medication because I did not believe in them.*…*they were not helpful whenever I used them. That's why I used to go to the hospital for medications, and that's what my mother was against.*
	Women's use of formal healthcare services may be discouraged by quality concerns; gender dynamics, including taboos in discussing intimate matters with male providers; and expectations of inadequate treatment.	*She* (female provider) *is different from the male doctor. The male doctor will tell the girl what he has read in the books, what is written, or what he tells her what he believes about menstruation. But for the female doctor, she's someone who has experience and has seen it for herself, also she has knowledge about menstruation*
Healthcare providers are underprepared to address menstruation and infibulation	Healthcare lacked training or guidance on both the distinct menstrual health concerns of those who have undergone infibulation.	*We don't have any guideline that we use as healthcare providers. We normally use the little knowledge that we know about FGM on the menstruation problem.*
	Providers lacked satisfactory treatment to address menstrual health complaints among infibulated women.	*Mostly*…*what we give them is pain management only*
	Confusion about the reach of Kenya's FGM/C ban was common among providers and undermined healthcare.	*…it (infibulation) is not allowed. You will be scolded and arrested for it*

### 3.1 Informal discussion shapes menstruation and infibulation knowledge

Regardless of the form of FGM/C they had undergone, nearly all women described how confusion, fear, and a lack of support shaped their experiences of both menstrual onset and of FGM/C. Many described the two events, FGM/C and menarche, as occurring close together in time during childhood and early adolescence. Most women reported receiving little to no information or guidance on menstruation before menarche, but nearly all described learning more about the topic during adolescence and adulthood, largely through stories or instructions from other women. This included information about managing menstruation and understanding their menstrual symptoms. They also learned about expected differences in menstrual symptoms between girls and women with firuani and sunni FGM/C. Although women questioned some of what they were taught by their elders, they appeared to develop a shared expectation that infibulation created distinct menstrual health problems. This showed up in how the women described their own or others' symptoms during interviews. In addition, some drew on this narrative in discussing community debates about the acceptability of firuani vs. sunni FGM/C and, specifically, as a reason to oppose firuani.

With very few exceptions, women remembered experiencing FGM/C between the ages of 7 and 12 years old. Mothers, grandmothers, and other female elders were described as deciding when and what form of FGM/C to perform, and then going on to perform the procedure, or turning to a traditional healer to do so. Some women participants spoke about learning that circumcision was a positive status in society, and thus had looked forward to the event, while others reported having no advance knowledge. Regardless, nearly all of the women interviewed shared how the procedure itself came as a shock, followed by pain and confusion. As one woman described:

There is no painkiller…after [the procedure] our mother carried us and she brought us to our home. Then they isolate us in one place—me and my sister…I don't remember how many days it was [that my legs were tied together] but more than one week we were there. (age 32, married)

Compounding their distress, some women referenced feeling alarm at how the adults involved in the procedure responded to their discomfort, pain, and fear. This included stories of some elders who, possibly fearing prosecution, had declined to seek formal healthcare for severe complications that a girl had experienced, such as excessive bleeding. Instead, adults used traditional remedies after the FGM/C procedure, such as herbs, to reduce pain or ward off infection. Many women further recalled receiving little emotional support, with female elders dismissing their reports of post-procedure pain by explaining that it was normal and should be endured without complaint.

Nearly all women described learning little or nothing about menstruation prior to menarche, which for most participants occurred between the ages of 11 and 15 years old. This resulted in alarm at the sight of their first menstrual blood. While mothers, sisters, and aunties provided practical menstrual guidance, this was typically only after their menstrual cycle had begun. One 30-year-old married woman remembered telling her mother that she thought she had been “pricked by a thorn,” which in turn did prompt an explanation. Some participants also reported schools providing minimal menstruation management guidance or information about the biological process of menstruation, but later in adolescence. A few also reported schools providing disposable menstrual pads, which were presented as preferable and more sanitary than cloth for managing blood flow.

In general, participants characterized informal guidance from female relatives and community women as their primary source of knowledge about menstruation. Much of this learning centered on how girls' lives changed once they reached menarche. As one 19-year-old single woman described, “When someone gets her period, they're automatically considered an adult”. This carried expectations that girls cover their hair (adopting hijab) and take on new household responsibilities, while also creating new family concerns about the potential for pregnancy. One 30-year-old married woman explained how families increase control of post-menarche girls' movements because she “has to be protected” from men. Many women also described mothers paying close attention to whether their daughters were menstruating regularly. A few noted that when mothers observed their daughters had not reached menarche by middle adolescence, or had irregular menstruation, they sought medical intervention. Thus, informal learning processes shaped women's understandings of menstrual health, including age of menarche and the potential fertility implications of irregular periods. The amount of women's knowledge, however, appeared to depend on having access to a female family member or friend who was knowledgeable and willing to discuss the topic.

Family members, friends, and other women were similarly found to influence women's understandings of the relationship between infibulation and menstrual health. Specifically, elders and friends conveyed that infibulation obstructed blood flow, through stories about their own and others' experiences. In this shared narrative, the women perceived such blockage to be caused by multiple factors, including excessive pain, and irregular flow or cycle length. As a 35-year-old married woman recalled hearing from traditional healers: “So as long as you're menstruating, you always experience the pain, there is a restriction [obstruction] that makes it not come out”. Another woman (age 25, single) reported that on reaching adolescence: “I was told that I had undergone the cut and the [menstrual] problems that are faced then [as a result]”. While she did not elaborate, she presented this as a distinct condition facing infibulated women. Other women suggested that older women might even intentionally withhold information about a connection between infibulation and painful menstruation to dismiss concerns about infibulation. One woman suggested that mothers believed that infibulation caused menstrual pain, but would deny this link to their daughters:

[The daughters] asked [their mothers] what could be the cause of those [menstrual] pains…Maybe they know the cause of the period pains is due to circumcision, but they won't tell you…they tell you it is normal. [woman, 23 years old, single]

She continued on to provide a much more satisfying explanation from a healthcare provider:

I remember a lady [female health provider] telling me that the wound had closed up further [after infibulation] and if she could cut it open…it would feel better. I had a feeling the period did not have an opening to come out from, and all the filth remained inside me.

The provider's explanation resonated with the participant's physical sensation that her body was constraining flow. Her idea of menstrual blood as “filth” indicated both negative perceptions of menstruation, and her sense of urgency in having the blockage released.

Several women also invoked the idea that infibulation obstructed menstrual flow as a reason to oppose the practice. This included for some an explanation of how their communities were moving toward the “sunni” form of FGM/C for health reasons. One woman drew a stark contrast in menstrual experiences between those who had and had not undergone infibulation: “people say the traditional way of cutting…the girls [were] disturbed during menstruation, some girls faint…but for sunni girls…menstruation is not disturbing*.”* (woman, age 32, married). She further suggested that the practice of infibulation is declining as communities became more aware of its health consequences, including those relating to menstrual health.

### 3.2 Multiple factors shape care-seeking for menstrual concerns

Women and healthcare providers generally agreed that menstrual health concerns were widespread among infibulated women, but that only a small proportion who experience severe menstrual-related symptoms seek care in formal health facilities. Factors that emerged as shaping women and girls' healthcare seeking behavior included women and family members' perceptions of symptom severity; their views of formal vs. alternative sources of care; household decision-making dynamics, and their perceptions of the quality of available care. Additional important influences included the gendered dynamics around patient-provider interactions, and those impacting household decision-making.

Both women and providers agreed that adolescent girls were much more likely to use formal health services for menstrual problems than adult women. However, participants had varied views on whether this indicated an actual difference in need. Several healthcare providers and women suggested that menstrual challenges would be resolved through sex and childbirth, either naturally, or because of a surgical procedure. As one 32-year-old married woman suggested, medical interventions were known to ease menstrual-related difficulties, which in turn would occur as an adolescent girl entered aspects of adult life:

They [adolescent girls] needed to be taken to the hospital and given some medicines [for menstrual pain]. Even when married they feel a lot of pain until they are taken to hospital. They have to make the hole big, the thing that sewed is cut…then those girls can feel a big change…the first time [they] get menstruation [they have pain] because the menstruation does not come out easily… but after they are cut [for childbirth] the menstruation comes out easily.

However other women dismissed the idea that menstrual problems subside after experiences that would presumably alter their infibulated status, such as marriage/sexual intercourse and childbirth:

There are those [infibulated women] who after being married they have menstruation every month. There are those [who] even after giving birth it is still painful…their bodies are returning to the circumcised way; it stays with them…If they go to the hospital [to give birth] the problem is still the same. (age 30, married)

Several providers also suggested that even if far more women who had been infibulated had a menstruation-related need than sought care, there were still significant numbers of women who showed up in their practices. Some estimated they saw multiple women each day with infibulation-related complaints, including menstrual health problems, infection, difficulty with sex, and obstetric care.

One common healthcare seeking pattern for women included over-the-counter medications. Several described using pain analgesics, procured at the local pharmacy or drug seller, as a common strategy for managing menstrual discomfort, regardless of FGM/C type. However as one male medical officer (age 30) explained, this was not always sufficient to meet the pain reduction needs of women with Type III FGM/C:

The common drug they use is buscopan [antispasmodic medication]…they self-medicate. Once the pain of the problem they're experiencing cannot be solved by the over-the-counter drugs, that is when they usually come to the hospital.

Not all women, however, pursued the hospital pathway. One woman (age 26, married) described those with Type III FGM/C as “becoming sick people,” with debilitating pain, who relied on pain medication “from the chemist” even though it did little to solve the problem. In her view, formal healthcare was meeting the women's needs, leaving them no suitable options.

Several women and providers described traditional remedies as a common part of care-seeking practices, although both groups of participants generally saw them as inadequate to meeting women's needs. Although the interviewed formal healthcare providers typically emphasized how inadequate such approaches were, the traditional birth attendant participant described giving those who had been infibulated black tea to “increase the flow” of menstrual blood. Several of the women expressed doubts about the value of traditional remedies, drawing a contrast between themselves and older women in their families. Disagreements over the value of traditional medicine were in fact found to be a source of tension in intergenerational female negotiations over sources of local care. While women described how their mothers or grandmothers believed in such healers, the participants described rejecting it for themselves and/or their daughters. As one 23-year-old single woman explained:

I wasn't getting better with those herbal medications because I did not believe in them…they were not helpful whenever I used them. That's why I used to go to the hospital for medications, and that's what my mother was against.

Beyond conflicts over whether to use traditional or formal health services, women generally characterized family involvement in care as a given. Adult female relatives appeared to play a central role in deciding when adolescent girls should get treatment for menstrual problems, and accompanied women to healthcare providers into adulthood. As one woman explained, it was impossible for women to seek care independently even after they were married: “If your husband is with you, he will talk on your behalf. But a girl alone? I have not heard of that, and it is not possible.*”* (age 26, single).

Several healthcare providers identified families' presence with girls and women as a barrier to providing quality care in formal settings. They perceived family members as adding to the challenge of communicating around menstruation and other topics related to sexual and reproductive matters. As one provider characterized, girls and women as “ready to talk, and we usually get information from them…the difficulty becomes when the lady or the child was brought by their parents, so we completely depend on the history given by the parents.” (male medical officer, age 30). In his view, parents would provide inaccurate descriptions of the problems. He speculated that this was because families did not communicate openly about intimate matters internally. Similarly, a female nurse (age 27) recounted how a woman came in with her aunt who explained that the woman was complaining of diarrhea. Once the aunt left the room, however, the patient explained that she was actually suffering from painful periods. In the nurse's view, this story illustrated how family members speaking for women could compromise confidentiality, and lead to misdiagnosis.

Women and providers alike agreed that norms discouraging women from discussing intimate matters with men sometimes inhibited dialogue in healthcare settings, including around menstrual health. One male gynecologist characterized Somali cultural taboos around women discussing genitalia with men. This led patients to use “code names” to refer to their anatomy and menstrual health concerns with him and other male providers. Women, however, described a more complex picture, with one 23-year-old single woman explaining that a female provider's lived experience with menstruation was reassuring for women who had undergone infibulation:

She [female provider] is different from the male doctor. The male doctor will tell the girl what he has read in the books, what is written, or what he tells her what he believes about menstruation. But for the female doctor, she's someone who has experience and has seen it for herself, also she has knowledge about menstruation

For many of the women, the prospect of discussing menstruation with any man was a challenge, as one 29-year-old woman explained: “It depends on how brave you are…some people fear males”. Another woman suggested that rather than women's beliefs or hesitation, the problem was “arrogant” male doctors who, “are not asking you about your concerns…they don't ask you or break it down for you” (age 35, married). However, she continued, “some doctors are good, and others are bad”. Such responses suggest that while gender or cultural norms might discourage women from seeking care or discussing concerns with male providers, provider communication skills and attitudes were also critically important.

### 3.3 Healthcare providers are underprepared to address menstrual health concerns among women who have undergone infibulation

Most of the formal healthcare providers emphasized a commitment to reducing the health burden of FGM/C, including what they perceived to be heightened infibulation-related menstrual health risks, but many also described having a lack of training or resources. Providers reported receiving limited guidance or education on either treating women with infibulation or addressing menstrual health concerns. Thus, they improvised diagnosis and treatment based on the skills and resources available to them. Uncertainty about their obligations under the Kenya FGM/C ban presented a further challenge for their delivery of care to this patient population.

Most providers reported that they and other healthcare workers had received very limited training on how to care for women who had undergone FGM/C. Among providers holding clinical roles with more advanced training, specialized care during childbirth was described as particularly relevant for patients who had undergone infibulation. The topic was otherwise primarily mentioned as a form of sexual and gender-based violence. There was no mention of guidance received around how to specifically approach the diagnosis, counselling, or treatment of menstrual health concerns among women with FGM/C, particularly for those who had been infibulated. Thus, care was delivered on what one nurse characterized as a “case by case” basis:

We don't have any specific guideline [for menstrual issues] because they come directly to seek medical healthcare, and then it is there where you start directly, because you know this is a case of FGM. We don't have any…training. (male, age 34)

Several providers stated that insufficient training and support left them underprepared to address menstrual health problems that they perceived as specific to infibulation. As one female nurse (age 27) characterized her own efforts and those of her colleagues: “We don't have any guideline that we use as healthcare providers. We normally use the little knowledge that we know about FGM on the menstruation problem”. Other providers drew on what they knew about infibulation to speculate on potential biological explanations for the pain and feelings of blocked menstrual flow that their patients reported:

This person, the blood is supposed to come out for, let us say, 2 days or 3 days. This blood is held inside because the space to come out is small and there's not a space to flow through since it has a scar. (male nurse, age 35)

In a few cases, providers' responses suggested they relied on an assumed association between infibulation and menstrual health complaints. For example, a female nurse (age 30) described how she approached diagnosis:

Okay, if a patient tells you this: “I have menstruation period, a painful one.” You ask them if they have undergone FGM, which type? They tell you. Now you automatically know this is the side-effect of [infibulation].

She further asserted that women who had experienced Type III FGM/C were bound to experience menarche at “age 21”. The first statement suggested that she, like women in the study, believed the presumed association between infibulation and menstrual health problems. However, the assumption of delayed menarche appeared at odds with what women reported about their age of menarche. As such, she illustrated how improvised explanations of the relationship between infibulation and menstrual health problems might lead providers to overattribute reported problems to infibulation. Other providers also grappled with the common expectation that infibulation-related menstrual health problems would resolve after marriage and childbirth. Some endorsed this view and suggested that there was little to do for girls reporting menstrual problems other than wait for the issue to resolve. A number of providers, however, classified this as a common but inaccurate “Somali belief”. According to one 30-year-old female nurse, many women would still seek menstrual pain relief after childbirth:

When you ask a mother who has delivered seven kids, for example, she normally tells you she has the same pain…No it [vaginal opening] does not open up…Because the FGM [infibulated status] is still the same. So the mother still experiences the same [menstrual] pain [as] when she had no baby.

The variation in providers' views reinforced the need for additional clinical training with a specific focus on the relationship between infibulation and menstrual problems. Similar to women participants, providers generally appeared to believe infibulation as impacting menstrual flow and experiences of pain. However, the absence of clear evidence on what clinical pathways this might represent hindered their ability to diagnose and treat reported problems.

Along with describing challenges around diagnosis of symptoms related to the intersection of menstruation and infibulation, the majority of providers identified few suitable treatment options. As a 34-year-old male nurse reported, “Mostly…what we give them is pain management only”. Yet he felt this was inadequate, as some women returned monthly for pain relief, and he and his colleagues could not offer any meaningful, lasting intervention. Many providers also described how resource constraints and other strains on the broader health system contributed to a view of menstrual health as a lower priority than more urgent issues related to infibulation, such as childbirth complications. Some did suggest that there were opportunities to improve care within existing health system constraints, with one female nurse (age 27) recommending a: “written guideline that will be fixed to the wall so that the other healthcare workers can use it when they are managing a patient with such issues (menstrual difficulties)”. She suggested that this format was necessary for those working busy facilities with a high workload, although it was not clear what such guidance should specifically include.

Kenya's FGM/C ban was also an important factor influencing providers, where it appeared mostly to sow confusion. While the traditional birth attendant expressed support for the practice of FGM/C, formal healthcare providers stressed opposition to the practice. Along with indicating their support for the ban, they demonstrated varying views of its practical application, and, by extension, what it required of them. For example, a male nurse (age 34), expressed a desire to secure punishment for people who participated in infibulation, while also questioning the value of punitive measures for changing a deeply engrained social practice. This pointed to a broader dilemma around whether the law required them to treat patients' family members as perpetrators of violence, educate them about risks of continuing the practice, or to focus on reducing harms already underway.

Women referenced two common and contrasting provider practices that further underscored the uncertainty surrounding the FGM/C ban. Several women expressed or referenced their concern that providers would punish women who sought care for infibulation-related concerns. As a 26-year-old married woman described, women might be taken to a health facility, but “…it (infibulation) is not allowed. You will be scolded and arrested for it”. This belief appeared to be a potential deterrent for women who might otherwise seek care. Notably, women classified this threat as solely applying to infibulation, even though the ban applies to all forms of FGM/C. In contrast, a few women also referenced their awareness of surgical interventions performed by local healthcare providers as important for addressing infibulation-related challenges negatively impacting sexual intercourse or menstrual flow. However, providers did not address this practice. Instead, they characterized pain relief as the only intervention they could offer to women with infibulation-related menstrual complaints.

Uncertainty around how to balance their personal investment in ending FGM/C with treating women's complaints was evident in providers' recommendations about how to handle menstrual health challenges among infibulated women. Surprisingly, no providers expressed interest in learning new clinical or counselling skills to treat menstrual problems, focusing their responses instead on infibulation. For example, several commented that they wanted to learn how to use their knowledge of health risks to persuade their patients to not cut their daughters, while others expressed an interest in promoting and/or performing defibulation.

## 4 Discussion

This exploratory qualitative study addressed the intersection of menstruation and infibulation among Somali women living in Kenya. Overall, findings situated reported infibulation-related menstrual health concerns, such as pain and irregular menstrual flow, within a social, structural, and policy context that may limit effective diagnosis and treatment of menstrual health conditions. In the absence of formal menstrual health education, informal learning processes among family and community members shape women's understanding and interpretation of a connection between infibulation and menstrual symptoms such as pain and irregular menstrual flow. Further, numerous factors influence the menstrual-related care seeking behaviors of women in this context, including those who have experienced infibulation. In addition, healthcare providers are underprepared to address both infibulation and menstrual health. These insights add to the still limited evidence suggesting that infibulation contributes negatively to menstrual health (4, 39, 40). Women's shared understanding that infibulation contributes to worse menstrual health, highlighted a need to invest in timely education for girls and women, improve the quality of clinical care for their experiences of menstrual discomfort, and build a supportive social and physical environment (23).

Our findings showed how women's experiences of menarche and FGM/C occurred in close succession. Both were marked by a lack of preparation, and feelings of confusion and distress. This was not surprising given that in the absence of formal menstrual education and support, girls in contexts around the world, including elsewhere in East Africa, have described their menstrual onset as upsetting and shameful (41–43). Similarly, as others have found feelings of distress, pain, and alarm are commonly expressed by Somali women who have undergone FGM/C, regardless of the type experienced (26, 44, 45). In women's telling, elders frequently dismissed such early experiences of distress and pain as “natural” and to be endured, perhaps referencing a documented sociocultural norm prizing Somali women's ability to endure pain without complaint (27). However, rather than prompting women to accept pain, this silencing of their experience seemed instead to produce frustration. In some cases, it raised doubts about their female relatives' guidance on menstrual health more broadly. Along with ensuring that girls have access to quality formal menstrual health education before reaching menarche, such insights highlighted the need to better understand and address female caregivers' own beliefs related to both menstrual health, FGM/C, and how they may intersect (2, 13, 14, 43, 46).

Women drew on stories, gossip, and other communications as informal learning processes that were critical sources of information and interpretations of the relationship between infibulation and menstrual health. Notably, the role of informal learning appeared to diverge somewhat from studies that suggest Somali women simply do not discuss menstrual health or other sexual and reproductive health topics (43). However, it did align with recent research on FGM/C practices in Somali communities suggesting that perceived contrasts in the health implications of firuani and sunni types are a major topic of discussion among women (13, 44). This included a specific shared narrative that infibulation led to “obstructed” menstrual blood flow and caused women to experience severe pain and irregular periods. It was evident that such an understanding was a useful way for women who had undergone infibulation to describe their menstrual-related symptoms. As such, it leant important social context to evidence that women report pain and irregular period length after infibulation (3, 4, 27). Specifically, the use of this narrative presented an example of how women may draw on a familiar set of beliefs in articulating their health needs in everyday life and bring those into clinical encounters. As such, it suggested an opportunity to affirm women's own understandings of their symptoms in the process of developing interventions to improve the quality of their care (47, 48).

Women's and providers' views of healthcare-seeking practices reflected the important roles of sociocultural beliefs and norms in shaping menstrual health-related behaviors. This included the common perception that menstrual symptoms would resolve after infibulated women married and gave birth. Similar findings have been documented among Somali women previously, including a belief that marriage and childbirth will diminish whatever problems they have as a result of infibulation (45, 49). This also may overlap with common narratives dismissing menstrual pain and discouraging women from seeking care that exist independent of FGM/C. For example, in other East African contexts, the documented belief that pain medication produces infertility may discourage girls and women from seeking care in formal health facilities (50). In addition, intergenerational tensions around the value of traditional medicine as compared with formal medical care suggested that multiple, potentially clashing beliefs are present and may shape healthcare seeking (51). Our findings suggested that women's or girls' use of traditional medicine reflected the persistence of a belief among some of the women involved in healthcare decision-making believed in such sources. However, they also showed that this was not an absolute barrier to accessing formal healthcare services.

Our findings underscored the substantial gendered barriers that affected healthcare seeking behaviors and use. Providers described how both intra-familial power dynamics and Somali gender norms discouraged both formal health system use, and straightforward reporting of menstrual concerns. They saw misdiagnosis arising from family members describing the health concerns for girls or women, or taboo and stigma around certain topics shrouding the real health issues. The healthcare providers' views aligned with past research documenting the challenges that can arise in settings where family members speak for women and girls in sexual and reproductive healthcare (35, 47, 48). The women's perspectives, however suggested that a more complex set of dynamics was at work. Some women saw relying on family members as an important buffer between themselves and providers they did not trust. Providers' attitudes and communication styles were the more important obstacle to clear communication and accurate diagnosis. In addition, several women participants suggested that women and family members would feel comfortable with female providers, and with male providers who they perceived to be respectful and knowledgeable. This aligned with previous research from multiple global contexts documenting how providers who adopt condescending attitudes toward women due to their gender, ethnicity, or infibulated status can discourage women from seeking care for sexual and reproductive health concerns (15, 35, 48, 52).

The gaps that emerged around healthcare providers' level of preparation and skill to provide specialized medical care for both menstrual health and infibulation revealed overlapping challenges. Across high and low resource contexts, including in Kenya, a lack of dedicated training and support for providers to effectively manage sexual and reproductive healthcare, including in relation to menstrual health, for women with infibulation is common (12, 22, 27, 46, 48). Such weak training, financial resourcing, and support for respectful care for women who have experienced infibulation, becomes a common deterrent to those seeking care across contexts (17, 25, 26).

In part, the lack of training reflects a distinct challenge for menstrual health care and infibulation. There is a gap in evidence on what role physiological factors associated with infibulation, such as scarring, infection, and nerve damage, may play in range of menstrual health symptoms (4, 13, 39). This evidence gap may also hinder providers' ability to recognize and treat menstrual disorders with other causes when they occur among women who have experienced infibulation (26).

Providers' lack of clarity regarding their obligations under the FGM/C ban appeared as a potential challenge to their ability to deliver quality care. They were unsure about what interventions they should offer, and how to treat women and accompanying family members they may suspect of violating the law. This confusion signaled a need to build providers' knowledge of the law as part of strengthening their capacity to provide culturally sensitive care for women who have experienced infibulation (22, 35, 47).

The findings suggest that beyond challenges in addressing the distinct needs of women who have experienced infibulation, providers also face knowledge gaps regarding the diagnosis and treatment of menstrual disorders. It was not clear from providers' accounts whether they had either training or experience in caring for women experiencing severe menstrual pain or irregular bleeding in the general population. However, previous studies have shown that shortcomings in provider preparation, diagnostic tools, and treatment options are barriers to addressing menstrual disorders among girls and women in Kenya and globally (29–31, 53, 54). Women and girls who experience menstrual discomfort may also be deterred from seeking care by stigma, messages dismissing or normalizing pain (30, 31, 50, 55).

### 4.1 Implications for policy and practice

This study demonstrated a need to refine and strengthen policy and practice around both menstrual health and infibulation. In Kenya and other contexts where national policy endorses menstrual health education, schools may only provide limited content, leaving girls to rely on incomplete, inaccurate, or negative messages about this basic bodily function (41). For those who, like the women in this study, experience FGM/C and menarche in short succession, early and sustained education is particularly important, given the complexity of their lived social and physical experiences. In parallel, ongoing approaches aimed at reducing FGM/C should continue. Community education interventions have effectively used content on women's rights and health to achieve reductions in FGM/C (34, 43). Given that menstrual health concerns appeared salient to women's perceptions of infibulation and its health effects, incorporating content on menstruation into existing FGM/C education models would be beneficial.

Our findings highlight the need to strengthen healthcare provider competencies to address both menstrual health concerns more broadly, and related needs among women who have experienced FGM/C, including infibulation. Educational approaches may draw on lessons and content from programmatic efforts to develop tools for provider training in skills such as culturally sensitive communication (9, 17, 47). Existing interventions to engage women affected by FGM/C in designing sexual and reproductive health care may provide important learning lessons. Examples have included convening Somali women's advisory groups to contribute to person-centered healthcare approaches, which treat understanding and responding to women's perspectives on their own bodies as central to guiding care (52, 56). These examples may provide a useful starting point for improving menstrual health care. When adapting provider skills-building interventions, addressing key topics, such as their obligations around national FGM/C bans, and good practices for managing family involvement in care, is essential. Finally, given the need to strengthen healthcare capacity to address menstrual health disorders across sub-Saharan African countries (53), specialized care for women who have undergone infibulation in relation to their menstrual health should be integrated into broader systems strengthening efforts.

### 4.2 Limitations

There are a few limitations to note. One, the research team did not screen participants for infibulation due to the sensitivity and illegality of FGM/C. This may limit a deeper understanding of the distinct menstrual needs and experiences of those who have undergone infibulation as compared to those who have experienced other forms of FGM/C, and those who have not experienced any form of FGM/C In addition, women who understood Kenya's FGM/C policy, and knew they could not be prosecuted for experiencing FGM/C may have been more likely to participate than those who were afraid to speak openly. Future research that specifically explores such experiences would prove useful. Two, providers represented various ethnic and linguistic backgrounds and professional training, and the three with the highest professional training were men. This may have shaped overall perspectives on menstrual health and infibulation, and the roles of rank and gender in shaping their perspectives may have been obscured. Future studies may seek to intentionally recruit providers based on gender, age, and training or rank to more systematically document various perspectives on care.

## 5 Conclusion

These findings illuminate the complex intersections between menstrual health and Type III FGM/C, or infibulation among Somali women living in Kenya. Multiple recommendations emerged, including the need for more research on the lived experience and biological dimensions of infibulation, symptoms, and effective healthcare responses across contexts. Clinical evidence on the physiological relationship between infibulation and menstrual health concerns would be particularly valuable. Educational interventions are needed to address the gap in education and support provided to girls and women around both menstrual health and their distinct concerns related to infibulation and FGM/C. Finally, tailored guidance is needed to improve the quality of healthcare delivery among providers caring for women who have undergone infibulation, on menstrual health more broadly. Overall, the study highlighted the importance of the importance of capturing women's voiced experiences of their lived realities around the intersection of menstrual health and infibulation.

## Data Availability

The data presented in this article are not available because the institutional review boards did not approve their release. Please contact Marni.Sommer@columbia.edu for data access queries.
